# Elucidation of antimicrobial activity and mechanism of action by *N-*substituted carbazole derivatives

**DOI:** 10.1016/j.bmcl.2017.08.067

**Published:** 2017-10-01

**Authors:** Johannes D. Clausen, Lasse Kjellerup, Karen O'Hanlon Cohrt, John Bondo Hansen, William Dalby-Brown, Anne-Marie L. Winther

**Affiliations:** a Pcovery ApS, Ole Maaløes Vej 3, 2200 Copenhagen N, Denmark; b Department of Plant and Environmental Sciences, University of Copenhagen, DK-1871 Frederiksberg, Denmark

**Keywords:** ATPase, Antifungal, Inhibitor, Membrane protein, Proton pump, Yeast, Bacteria, Carbazole

## Abstract

Compounds belonging to a carbazole series have been identified as potent fungal plasma membrane proton adenosine triphophatase (H^+^-ATPase) inhibitors with a broad spectrum of antifungal activity. The carbazole compounds inhibit the adenosine triphosphate (ATP) hydrolysis activity of the essential fungal H^+^-ATPase, thereby functionally inhibiting the extrusion of protons and extracellular acidification, processes that are responsible for maintaining high plasma membrane potential. The compound class binds to and inhibits the H^+^-ATPase within minutes, leading to fungal death after 1–3 h of compound exposure *in vitro*. The tested compounds are not selective for the fungal H^+^-ATPase, exhibiting an overlap of inhibitory activity with the mammalian protein family of P-type ATPases; the sarco(endo)plasmic reticulum calcium ATPase (Ca^2+^-ATPase) and the sodium potassium ATPase (Na^+^,K^+^-ATPase). The ion transport in the P-type ATPases is energized by the conversion of ATP to adenosine diphosphate (ADP) and phosphate and a general inhibitory mechanism mediated by the carbazole derivative could therefore be blocking of the active site. However, biochemical studies show that increased concentrations of ATP do not change the inhibitory activity of the carbazoles suggesting they act as allosteric inhibitors. Furthermore decreased levels of intracellular ATP would suggest that the compounds inhibit the H^+^-ATPase indirectly, but *Candida albicans* cells exposed to potent H^+^-ATPase-inhibitory carbazoles result in increased levels of intracellular ATP, indicating direct inhibition of H^+^-ATPase.

## Graphical abstract

**Figure:**
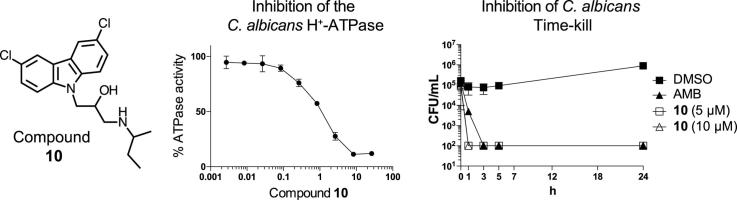

## Introduction

Each year, 2 million people contract an invasive fungal infection (IFI) worldwide, and with mortality rates reaching 95% depending on the pathogen and underlying risk factors, IFIs represent a significant public health problem.^1^ Despite a rising number of emerging invasive fungal pathogens, the majority of IFI’s are caused by the yeast *Candida albicans* and the mold *Aspergillus fumigatus*. Candidemia (caused by *Candida* spp.) is among the top 5 most common nosocomial blood-borne infections in the US.^2^ Individuals most at risk of developing life-threatening fungal infections are those on immunosuppressive therapies, aggressive chemotherapies, HIV-infected patients and those with congenital immunodeficiencies (e.g. chronic granulomatous disease).^3^ Continuous advances in surgical procedures, organ transplantation medicine and chemotherapeutic regimens, as well as ongoing resistance development coincides with an increase in the incidence of difficult-to-treat invasive fungal disease.^4^, ^5^ Current antifungal treatments represent 3 main compound classes; the polyenes (e.g. amphotericin B), the azoles (e.g. voriconazole, fluconazole), and the echinocandins (e.g. caspofungin). These current therapies collectively suffer from shortfalls such as toxicity, drug-drug interactions, narrow spectrum of activity, and resistance development. Furthermore, the emergence of multidrug-resistant *Candida* and *Aspergillus* isolates is an increasing concern.^6^ Besides the inherent limitations in the current compound classes, initiation of appropriate treatment is often delayed by challenges in diagnosis.^7^ Therefore, new classes of safe, well-tolerated broad-spectrum antifungal drugs without drug-drug interactions and a propensity for resistance are urgently needed.^8^

The fungal plasma membrane H^+^-ATPase is essential for fungal growth and survival.^9^ The H^+^-ATPase is a proton pump, generating the electrochemical gradient across the fungal plasma membrane by transporting protons from the cytoplasm to the extracellular site. This process is energized by the conversion of ATP to ADP and phosphate. Fungal plasma membrane proton pumps belong to the P_III_-type ATPase family,^10^ and the H^+^-ATPase is highly conserved across the fungal kingdom, with 80–90% sequence identity between the H^+^-ATPase in different *Candida* species. In mammalian cells, the functionally related P_II_-type Na^+^,K^+^-ATPase is responsible for maintaining the ion gradient across the plasma membrane.^11^, ^12^ The sequence identity of the H^+^-ATPase to the mammalian P_II_-type ATPases, Na^+^,K^+^-ATPase and the sarcoplasmic reticulum Ca^2+^-ATPase, is less than 30%. The activity of the Na^+^,K^+^-ATPase is targeted with cardiotonic steroids (CTS) in the treatment of congestive heart failure.^13^ The Ca^2+^-ATPase has been identified as a promising anticancer target using a pro-drug approach with the highly potent and specific Ca^2+^-ATPase inhibitor thapsigargin.^14^ The essential nature of the conserved fungal H^+^-ATPase, which is absent from mammalian cells, makes it an attractive target for the development of novel broad-spectrum antifungal agents.

**Fig. 1:**
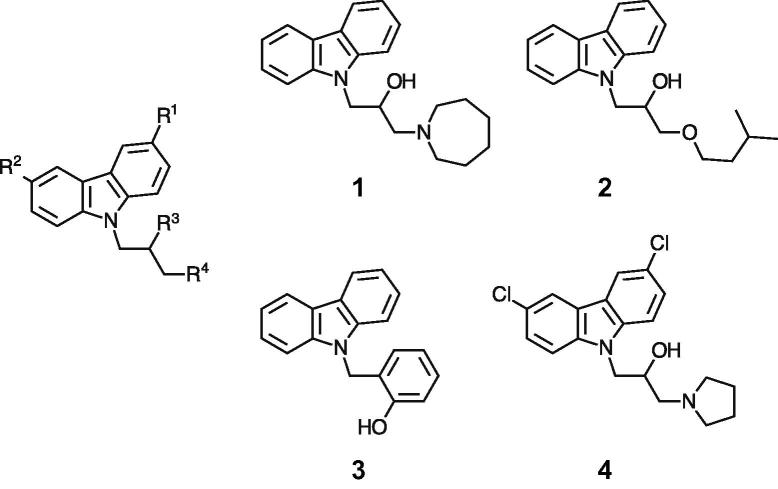
Carbazole scaffold (left) and structures of initial H^+^-ATPase inhibitor hits **1**–**4**.

In the search for novel H^+^-ATPase inhibitors, a library screen of 20,240 small molecule compounds was conducted by screening for H^+^-ATPase inhibitory activity at a compound concentration of 20 µM. Four compounds containing an *N1*-substituted carbazole moiety were identified from the library screening as novel H^+^-ATPase inhibitors (Fig. 1), and the ATP hydrolysis IC_50_ was determined together with antifungal activity against *S. cerevisiae* and *C. albicans* (Table 1). Compound **4** was the most potent antifungal compound, which displayed H^+^-ATPase inhibitory activity.

**Table 1:** IC_50_ determination of the H^+^-ATPase inhibition and minimal inhibitory concentration determination (MIC) of fungal growth by initial hit compounds **1**–**4**.

	ATP hydrolysis IC_50_ [µM]	Fungal Growth Inhibition MIC [µM]	
	*S. cerevisiae* H^+^-ATPase	*S. cerevisiae*	*C. albicans*
1	18.8 ± 7.3	>200	>200
2	5.5 ± 0.7	50	>200
3	9.7 ± 0.6	50	100
4	17.3 ± 5.5	10	30

**Fig. 2:**
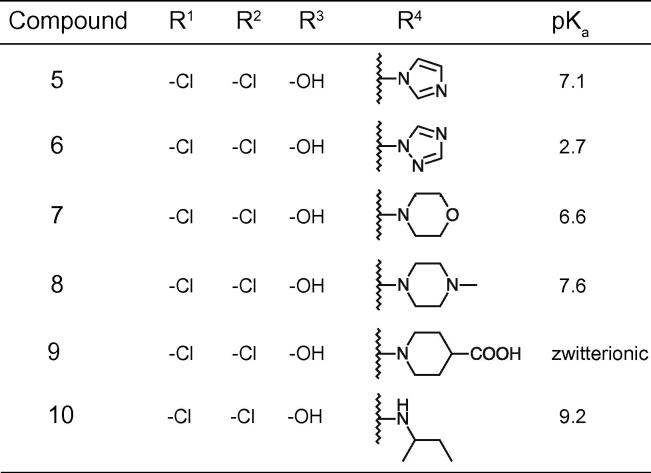
Structures of compounds **5**–**10**.

**Fig. 3:**
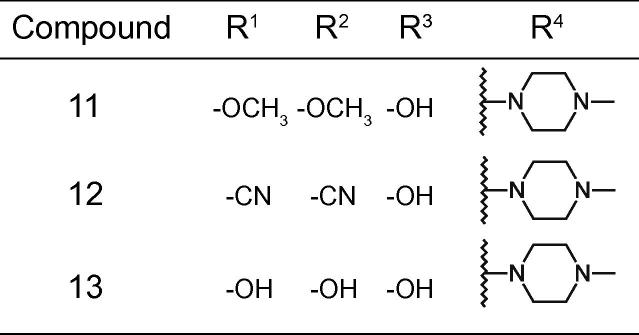
Structures of compounds **11**–**13**.

**Fig. 4:**
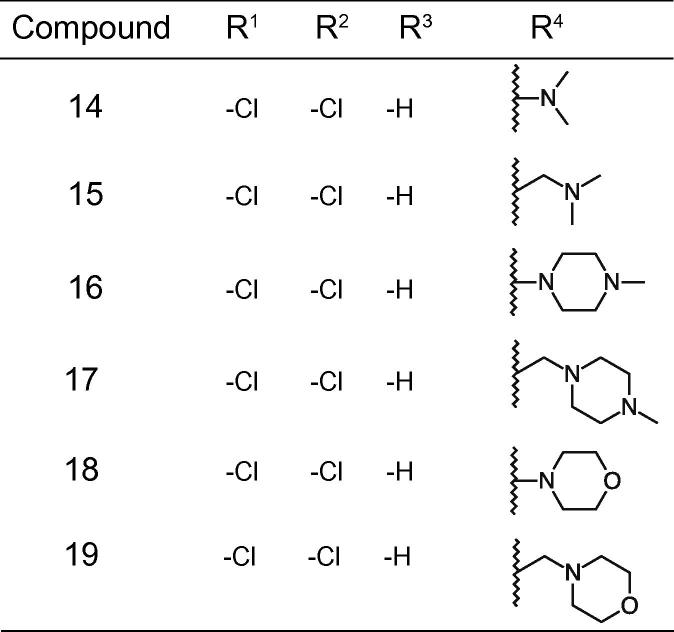
Structures of compounds **14**–**19**.

It seemed plausible from the limited structure-activity relationship available that the chloro substitutions of R^1^ and R^2^, possibly in combination with some size exclusion in R^4^, were driving the cellular activity (comparing compound **4** with **1** and **2**, and compound **4** with compound **3**, respectively), given that the ATP hydrolysis IC_50_ was similar for all four compounds. Based on this hypothesis fifteen compounds were synthesized to further explore the structure-activity relationship (Fig. 2, Fig. 3, Fig. 4). The compounds were characterized for H^+^-ATPase inhibition and antifungal activity by means of an ATP hydrolysis assay and a fungal growth inhibition assay, respectively. Furthermore a study was conducted to investigate if the binding site of the compounds overlapped with the nucleotide-binding site within the H^+^-ATPase, and to investigate possible effects on the intracellular ATP level in *C. albicans*. Additionally, the ability of selected compounds to inhibit acidification of the surrounding media of fungal cells after addition of glucose was investigated. Finally, we determined whether the compounds acted in a fungistatic or a fungicidal manner.

To investigate the H^+^-ATPase inhibitory effect of the compounds we isolated plasma membranes containing H^+^-ATPase from *S. cerevisiae* and *C. albicans* cells.^15^ Plasma membranes isolated from pig kidney as well as endoplasmic reticulum membranes from rabbit hind leg muscle were used to counter-screen compounds for activity against mammalian Na^+^,K^+^-ATPase and Ca^2+^-ATPase, respectively. Prior to their use, fungal plasma membrane batches were validated by determining the maximum ATP hydrolysis activity, the pH optimum of the ATPase activity, and the sensitivity to inhibition by orthovanadate. ATPase activity was determined by a colorimetric assay that measures the amount of inorganic phosphate liberated over time, as described in Kjellerup et al.*,* 2017.^15^ Table 2, Table 3, Table 4 list the IC_50_ values obtained by measuring the concentration dependence of the ATPase activity for the fifteen carbazole compounds with each of the four membrane preparations.

**Table 2:** IC_50_ determination of ATPase inhibition and MIC (MFC) determination of fungal growth of initial hit compounds **5**–**10**.

Compound	ATP hydrolysis^a^ IC_50_ [µM]				
	S. cerevisiae H^+^-ATPase	C. albicans H^+^-ATPase		Mammalian Ca^2+^-ATPase	Mammalian Na^+^,K^+^-ATPase
5	23.0 ± 12.3	19.3 ± 2.6		10.7 ± 2.2	20.4 ± 2.3
6	7.4 ± 1.6	4.0 ± 1.2		20.3 ± 3.6	5.2 ± 0.3
7	6.9 ± 1.3	2.6 ± 0.4		19.6 ± 7.2	6.0 ± 1.3
8	52.7 ± 10.6	26.4 ± 2.5		36.8 ± 12.2	9.7 ± 3.3
9	106.8 ± 11.8	105.3 ± 13.9		97.6 ± 25.7	4.0 ± 0.7
10	2.0 ± 0.2	1.1 ± 0.2		0.3 ± 0.1	1.0 ± 0.2

	Fungal Growth Inhibition MIC (MFC) [µM]				

Saccharomyces cerevisiae ATCC 9763	Candida albicans SC5314	Candida krusei ATCC 6258	Candida glabrata ATCC 90030	Candida glabrata Cg003

5	24 (75)	24 (>75)	>75 (>75)	>75 (>75)	>75 (>75)
6	75 (>75)	75 (>75)	>75 (>75)	>75 (>75)	>75 (>75)
7	24 (24)	75 (75)	>75 (>75)	>75 (>75)	>75 (>75)
8	7.5 (7.5)	68 (75)	75 (>75)	42 (75)	58 (75)
9	>75 (>75)	>75 (>75)	>75 (>75)	>75 (>75)	>75 (>75)
10	0.4 (1.2)	3.7 (3.8)	2.9 (3.8)	1.2 (3.8)	5.6 (7.9)

a ATP hydrolysis data determined at pH 7 (*n* = 3).

**Table 3:** IC_50_ determination of ATPase inhibition and MIC (MFC) determination of fungal growth of initial hit compounds **11**–**13**.

Compound	ATP hydrolysis^a^ IC_50_ [µM]				
	S. cerevisiae H^+^-ATPase	C. albicans H^+^-ATPase		Mammalian Ca^2+^-ATPase	Mammalian Na^+^,K^+^-ATPase
11	>167	>167		>167	>167
12	>167	144.1 ± 14.7		92.7 ± 13.1	10.5 ± 1.4
13	>167	>167		>167	>167

	Fungal Growth Inhibition MIC (MFC) [µM]				

Saccharomyces cerevisiae ATCC 9763	Candida albicans SC5314	Candida krusei ATCC 6258	Candida glabrata ATCC 90030	Candida glabrata Cg003

11	>75 (>75)	>75 (>75)	>75 (>75)	>75 (>75)	>75 (>75)
12	>75 (>75)	>75 (>75)	>75 (>75)	>75 (>75)	>75 (>75)
13	>75 (>75)	>75 (>75)	>75 (>75)	>75 (>75)	>75 (>75)

a ATP hydrolysis data determined at pH 7 (*n* = 3).

**Table 4:** IC_50_ determination of ATPase inhibition and MIC (MFC) determination of fungal growth of initial hit compounds **14**–**19**.

Compound	ATP hydrolysis^a^ IC_50_ [µM]				
	S. cerevisiae H^+^-ATPase	C. albicans H^+^-ATPase		Mammalian Ca^2+^-ATPase	Mammalian Na^+^,K^+^-ATPase
14	36.2 ± 3.1	23.3 ± 5.3		13.0 ± 2.1	2.1 ± 0.5
15	51.8 ± 0.5	31.1 ± 4.8		17.5 ± 11.4	5.1 ± 0.8
16	20.0 ± 1.0	7.1 ± 0.5		23.2±6.4	2.5 ± 0.6
17	18.7 ± 2.4	15.4 ± 2.6		17.5 ± 2.1	4.7 ± 0.9
18	11.1 ± 3.8	5.9 ± 1.7		70.9 ± 8.4	7.7 ± 3.1
19	24.9±1.8	11.6 ± 1.6		95.4 ± 20.5	5.6 ± 0.5

	Fungal Growth Inhibition MIC (MFC) [µM]				

Saccharomyces cerevisiae ATCC 9763	Candida albicans SC5314	Candida krusei ATCC 6258	Candida glabrata ATCC 90030	Candida glabrata Cg003

14	5.8 (16)	24 (75)	13 (24)	24 (24)	24 (75)
15	2.4 (5)	24 (24)	7.5 (16)	7.5 (7.5)	7.5 (24)
16	2.4 (2.4)	24 (24)	7.5 (7.5)	7.5 (7.5)	7.5 (7.5)
17	2.4 (2.4)	24 (24)	7.5 (7.5)	7.5 (7.5)	7.5 (24)
18	>75 (>75)	>75 (>75)	>75 (>75)	>75 (>75)	>75 (>75)
19	7.5 (7.5)	>75 (>75)	>75 (>75)	24 (>75)	24 (>75)

a ATP hydrolysis data determined at pH 7 (*n* = 3).

Compared to the parent compounds **1**–**4**, compound **10** exhibited the highest potency for H^+^-ATPase inhibition, with IC_50_ values of 1.1 and 2 µM for *C. albicans* and *S. cerevisiae* H^+^-ATPase, respectively, i.e. up to ca. 9-fold more potent than the parent compounds. H^+^-ATPase inhibition was generally ∼2-fold lower for the *C. albicans* H^+^-ATPase than for the *S. cerevisiae* H^+^-ATPase. It should be considered, however, that this difference need not necessarily reflect a difference in the affinity of the compounds for the two fungal H^+^-ATPases, but may reflect different functional properties of the pumps themselves. Provided that the compounds inhibit the pumps by binding to a specific protein conformational state in the transport cycle (for P-type pumps this is commonly referred to as the E1-E2 cycle^16^), a lower IC_50_ would also be expected if that particular conformational state was more prevalent or long-lived in the cycle for one protein compared to the other. Such differences may come about by variations in the degree of post-translational modification levels.^17^ A similar argument can be made with respect to the differences in IC_50_ values between the fungal (P_III_-type) and the two mammalian pumps (P_II_-type). Nevertheless, the majority of the compounds do not exhibit selectivity for either fungal or the mammalian ATPases, inhibiting all four enzymes with similar IC_50_ values, with a few notable exceptions. For instance, **9** and **12** displayed pronounced selectivity towards Na^+^,K^+^-ATPase (i.e. lower IC_50_ value) and could be an interesting starting point in the pursuit of a specific Na^+^,K^+^-ATPase inhibitor (Table 2, Table 3). Additionally, **18** and **19** were much less potent on the Ca^2+^-ATPase than the three other pumps (Table 3). However, in general, the carbazole series shows little discrimination in its inhibition of P_II_- and P_III_-type ATPases. Initially, we attempted to explore variations in R^4^. Noting that the basic center in R^4^ was not a prerequisite for ATPase inhibitory activity (*c.f.* compound **2** and **3**), we prepared analogues of compound **4** with a range in pK_a_ (Fig. 2). Compound **10** was by far the most potent ATPase inhibitor and antifungal compound, supporting the notion that the basic moiety (compounds **4** and **10**) with an (flexible) apolar terminal chain *β* to the hydroxy group (compounds **6**, **7** and **10**) is preferred for optimal activity. The more drug-like compound **8**, with moderate ATPase and fungal inhibitory activity, was chosen as a template to explore the importance of substitutions in R^1^ and R^2^. It was already determined (compounds **1**–**3**) that substitution was not required for activity and that a lipophilic substitution could potentially increase antifungal activity. We decided to explore this notion more broadly by preparing analogues with small and highly polar substitutions capable of making hydrogen bonds (compounds **11**–**13**, Fig. 3). All three resulting compounds exhibited a complete loss of both H^+^-ATPase inhibitory activity and antifungal activity (Table 3).

Finally, we proceeded to investigate the importance of the alkyloxy group using the same piperazine template (compound **16**) as before. We discovered that we could either simplify the terminal apolar chain (compounds **14** and **15**) or increase the chain (compounds **17** and **19**) with no dramatic change in activity (Fig. 4, Table 4). In conclusion, an effort was made to explore the pharmacophore for the carbazole series, which revealed that polar substitutions at R^1^ and R^2^ resulted in loss of the H^+^-ATPase inhibitory activity, hydrogen at R^3^ improved H^+^-ATPase inhibitory activity as compared to a hydroxyl-group (comparing compound **16** with **8**), and substitutions of different length in R^4^ was allowed. Additional work could include ring opening of the carbazole core to decrease possible π-stacking (and aggregation), as well as trying to generate stronger interactions in the *N1*-chain in order to increase overall potency of the carbazole series and realize ATPase specificity for H^+^-ATPase.

**Fig. 5:**
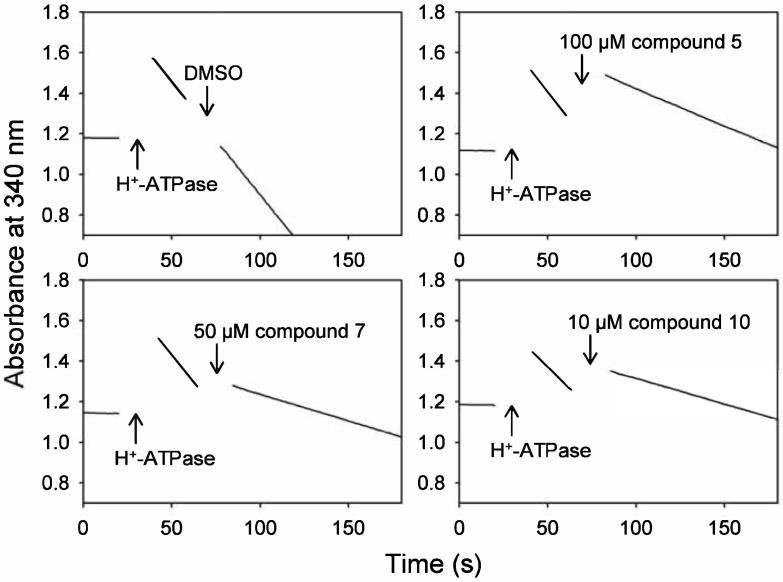
Kinetics of H^+^-ATPase inhibition by carbazole compounds. The initial rate of ATP hydrolysis catalyzed by *S. cerevisiae* H^+^-ATPase was determined by an NADH-coupled ATPase assay, as described in the Supporting Information. The reaction was initiated by the addition of H^+^-ATPase-containing membranes (arrows with “H^+^-ATPase”) to a pH 7 buffer containing 5 mM ATP. At the indicated times the respective compounds were added to give the final compound concentrations shown in the figure and a final DMSO concentration of 1.1%.

To investigate whether the ATPase inhibition observed was due to compound binding at the ATP binding site, three compounds (**5**, **7**, and **10**) were selected for further evaluation in an ATPase activity assay on *S. cerevisiae* H^+^-ATPase-containing membranes in presence of increasing ATP concentrations. The initial rate of ATP turnover was measured spectrophotometrically by an NADH-coupled ATPase activity assay that uses phosphoenolpyruvate to regenerate ATP, as described in Møller et al., 1980.^18^ Fig. 5 shows representative data from the spectrophotometric traces obtained with 5 mM ATP in the reaction buffer containing either DMSO or inhibitory concentrations of **5**, **7** or **10**. DMSO alone had no effect on the initial rate of ATP hydrolysis, whereas the three compounds significantly and rapidly reduced the rate of ATP hydrolysis.

**Fig. 6:**
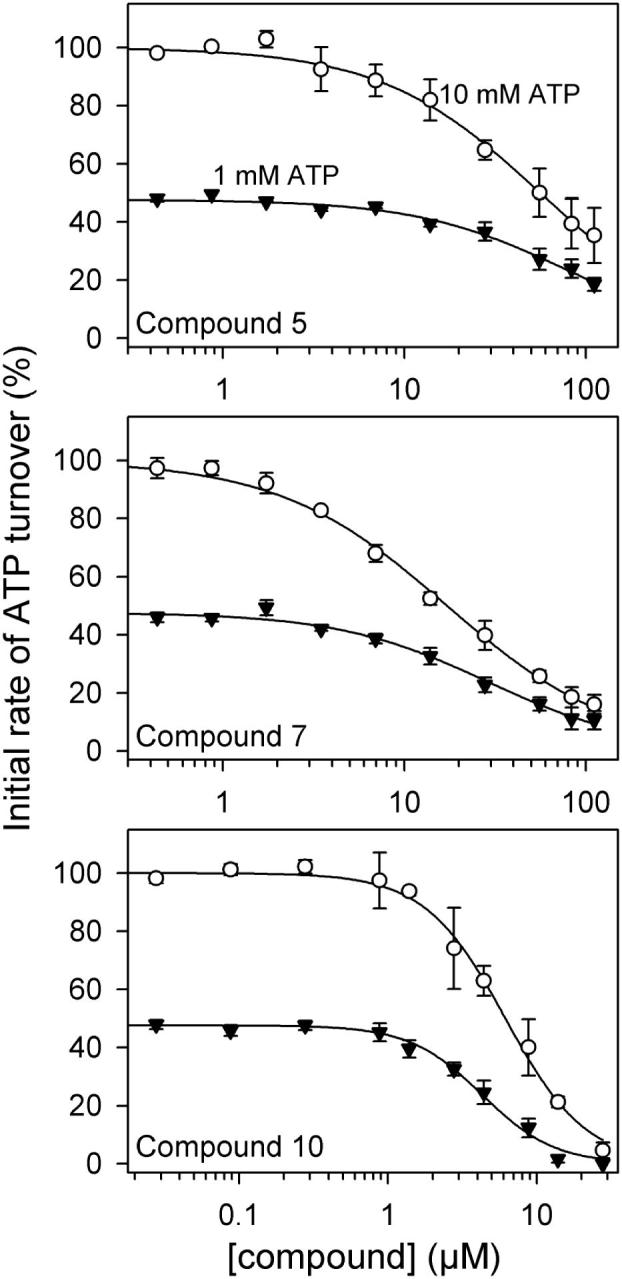
Compound dependence of the initial rate of H^+^-ATPase activity at 1 mM (*triangles*) or 10 mM (*circles*) ATP. The initial rates following compound addition deduced from experiments similar to those shown in Fig. 5 were normalized to the maximal rate obtained at 10 mM ATP in the absence of compound (presence of 1.1% DMSO) and plotted as a function of the compound concentration. The lines show the best fits of the equation V = V_max_ · (1 − [cpd]^h^/(IC50^h^ + [cpd]^h^)) to the data (*n* = 3), giving the following IC_50_ values: compound **5**, 1 mM ATP, 78.0 ± 14.9 µM; compound **5**, 10 mM ATP, 60.0 ± 14.2 µM; compound **7**, 1 mM ATP, 29.7 ± 6.7 µM; compound **7**, 10 mM ATP, 16.8 ± 1.7 µM; compound **10**, 1 mM ATP, 4.5 ± 0.5 µM; compound **10**, 10 mM ATP, 6.3 ± 1.2 µM.

In Fig. 6, the initial rate of ATP hydrolysis, obtained in similar experiments carried out in the presence of either 1 or 10 mM ATP, is plotted as a function of compound concentration. The maximal rate of ATPase activity in the absence of compound increased 2.1-fold (±0.3; *n* = 9) from 1 to 10 mM ATP, owing to the fact that 1 mM ATP is not saturating for the transport activity. The IC_50_ values for the compounds did not increase appreciably despite the 10-fold increase in ATP concentration, suggesting that the binding site for the compounds does not overlap with that of ATP at the nucleotide-binding site, and, hence, that the compounds act as allosteric inhibitors. Importantly, the three compounds display a similar relative pattern in the two ATPase assays, with compound **10** displaying the lowest IC_50_ and compound **5** the highest IC_50_.

We subsequently assessed the carbazole series for antifungal activity, as described in Kjellerup et al., 2017.^15^ Table 2, Table 3, Table 4, Table 5 list the minimum inhibitory concentrations (MIC) and the minimum fungicidal concentrations (MFC) of the fifteen compounds tested against the yeasts *Saccharomyces cerevisiae*, *Candida albicans*, *C. krusei*, and two isolates of *C. glabrata* (a wild-type strain ATCC 90030 and a clinical azole-resistant isolate Cg003 with increased efflux pump expression^19^), and the molds *Aspergillus fumigatus* and *A. flavus*. MIC was defined as the lowest concentration inhibiting visual growth of the microorganism in RPMI-1640 media. Compound **10**, the most potent ATPase inhibitor, was also the most potent antifungal compound, with MIC values ranging from 0.4 to 12 µM on all fungal isolates tested, in comparison to MICs of 30 µM and above against *C. albicans* for compounds **1**–**4**. Although **10** exhibited a 5-fold higher MIC against the azole-resistant than the wild-type *C. glabrata* isolate, it is encouraging to see activity against this isolate, despite its overexpression of the multidrug resistance efflux pumps Cdr1p and Cdr2p. A number of additional compounds (**14**–**17**) demonstrated greater antifungal activity against all yeast isolates (MICs ranging from 2.4 to 24), as compared to **1**–**4**. However, in several cases antifungal activity against molds was minimal, Table 5.

**Table 5:** Mold growth inhibition data (n=3).

Compound	Fungal Growth Inhibition MIC [µM]	
	*Aspergillus* *flavus* ATCC MYA-1005	*Aspergillus fumigatus* ATCC 13073
**5**	>75	>75
**6**	>75	>75
**7**	>75	>75
**8**	>75	75
**9**	>75	>75
**10**	12	3.7
**11**	>75	>75
**12**	>75	>75
**13**	>75	>75
**14**	>75	>75
**15**	75	24
**16**	24	41
**17**	24	24
**18**	>75	>75
**19**	>75	>75

**Fig. 7:**
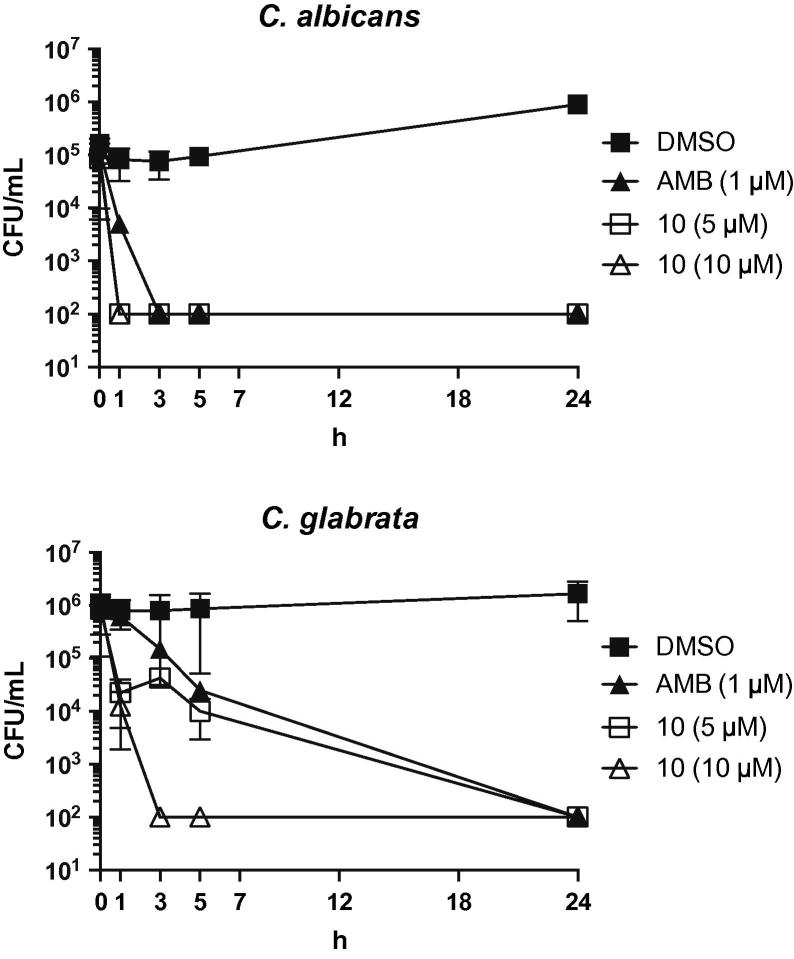
Time-kill data for *C. albicans* and *C. glabrata* cells (1 × 10^5^ CFU/ml) incubated with DMSO (final concentration 1%), 1 µM AMB, and 5 or 10 µM of compound **10**.

Interestingly, most of the compounds exhibited comparable MIC values for both *C. glabrata* isolates (Table 2, Table 4), suggesting that the carbazole compounds are generally less prone to efflux pumping than the azoles, such as fluconazole.^19^, ^20^ Time-kill studies were carried out to investigate whether the antifungal activity observed for **10** was fungistatic or fungicidal. Given the essential nature of fungal H^+^-ATPase, it is anticipated that compounds targeting H^+^-ATPase activity will promote fungicidal effects.^21^ Indeed, exposure of *C. albicans* or wild-type *C. glabrata* cells to **10** resulted in cell death comparable to that observed for amphotericin B (AMB), which is a known fungicidal compound (Fig. 7).

It was subsequently evaluated whether or not the carbazoles were able to prevent extracellular acidification of the surrounding media by blocking the pumping activity of the H^+^-ATPase within fungal cells.^15^ Compounds **10**, **16** and **17**, all of which were found to be potent enzymatic inhibitors of the H^+^-ATPase, were also potent inhibitors of proton export from fungal cells, while the control compound voriconazole did not affect proton transport (Table 6). We then assessed the compounds for potential effects on fungal intracellular ATP levels, which have previously been shown to increase upon reduced H^+^-ATPase activity.^15^, ^22^, ^23^, ^24^ The H^+^-ATPase requires ATP to transport protons out of fungal cells. Consequently, a decrease in intracellular ATP (iATP) levels will indirectly affect H^+^-ATPase activity. To determine if the H^+^-ATPase-inhibitory activity of the carbazoles was indirectly caused by a decrease in ATP levels, iATP levels were determined upon exposure to **7**, **8**, **10** and **15**–**17** (Table 6). In all cases, iATP levels were increased in *C. albicans* cells exposed to carbazoles compared to the untreated control. The most potent H^+^-ATPase inhibitor compound **10** resulted in the largest iATP increase, and the least potent H^+^-ATPase inhibitors **8** and **15** resulted in the lowest increase in iATP. Compound **7** was a potent inhibitor of the enzymatic activity of the H^+^-ATPase (IC_50_: 2.6 and 6.9 µM, Table 2), but was not very effective in preventing extracellular acidification from whole fungal cells and resulted only in slight increase in iATP. Compound **7** also displayed poor antifungal activity, similarly to the related compounds **18** and **19**, suggesting that the morpholino R^4^ substitution in **7**, **18** and **19** result in poor H^+^-ATPase inhibition within whole fungal cells. This implies that the carbazoles need to pass the fungal membrane to inhibit the H^+^-ATPase and **7**, **18** and **19** may have reduced membrane permeability. In summary, these data support our working hypothesis that the carbazoles inhibit the H^+^-ATPase in fungal cells, leading to reduced ATP hydrolysis and a concomitant increase in intracellular ATP.

**Table 6:** iATP and extracellular acidification IC_50_ data. Compounds **7, 8, 10, 15, 16, 17** and voriconazole tested at 20 µM for iATP.

Compound	iATP^a^ (nM)	Extracellular acidification^b^ IC_50_ (µM)	
	*C. albicans*	*S. cerevisiae*	*C. albicans*
DMSO	38 ± 17	NA	NA
**7**	93 ± 45	33.3 ± 0.6	22.2 ± 12.9
**8**	70 ± 25	11.1 ± 2.1	9.7 ± 6.8
**10**	491 ± 79^*^	2.1 ± 1.0	2.4 ± 1.2
**15**	53 ± 10	16.2 ± 2.6	13.1 ± 7.2
**16**	235 ± 70^*^	7.3 ± 0.4	9.5 ± 2.5
**17**	221 ± 54^*^	10.6 ± 4.5	9.0 ± 1.9
Voriconazole	37 ± 19	>75	>75

a Data show mean ± SEM with *n* = 5. One-way ANOVA to compare differences between iATP levels upon compound exposure and DMSO control (* >95% confidence interval).

b IC_50_ is determined as the concentration that results in 50% inhibition of the media acidification normalized to the response from glucose-activated versus non-glucose-activated cells. Data show mean ± SEM with *n* = 3. NA: Not applicable.

To further support the notion that the carbazole H^+^-ATPase inhibitors were specific inhibitors of fungal growth, we tested the effect of all fifteen compounds on the growth of two bacterial species; the gram-positive *Staphylococcus aureus* and the gram-negative *Escherichia coli*. The MICs for these 15 compounds are presented in Table 7. The only compound to exert potent low micromolar antibacterial activity on both *S. aureus* and *E. coli* was **10**, which from our ATPase activity experiments was shown to possess very potent broad-spectrum ATPase activity. Compound **16** and **17** were interesting as they were potent inhibitors of the ATP hydrolysis in the enzymatic assay and potent H^+^-ATPase inhibitors in whole fungal cell assay (iATP and extracellular acidification). Assuming that carbazoles bind from the cytoplasmic site to the H^+^-ATPase, these data indicate that **16** and **17** have good membrane permeability. Given the potent H^+^-ATPase-inhibitory activity of these compounds it is encouraging that they exhibited better antifungal than antibacterial activity, supporting the notion that H^+^-ATPase is a promising and unique target for antifungal drug development.

**Table 7:** Bacterial growth inhibition data (*n*=3).

Compound	*Escherichia coli* ATCC 25922	*Staphylococcus aureus* ATCC 29213
*Growth inhibition MIC [µM]*		
**5**	>75	7.5
**6**	>75	41
**7**	>75	41
**8**	75	64
**9**	>75	75
**10**	20	2.9
**11**	>75	>75
**12**	>75	>75
**13**	>75	>75
**14**	75	7.5
**15**	75	24
**16**	75	24
**17**	75	24
**18**	>75	>75
**19**	>75	24

In line with expectations from a fungal H^+^-ATPase inhibitor, we show here that carbazoles that potently inhibit the H^+^-ATPase also exhibit broad spectrum antifungal activity.^15^, ^21^ Furthermore, these compounds led to an increase in intracellular ATP levels and act in a fungicidal manner, as evidenced by MFC and time-kill data. An extensive number of carbazoles with a very diverse set of substitutions were previously reported to exhibit large variations in their biological activities.^25^, ^26^, ^27^, ^28^, ^29^ Of particular interest is that certain carbazoles, displaying a similar scaffold substitution to those reported in this article, have previously been reported as antifungal agents, but with no report on the antifungal mode of action.^25^ The halogenated carbazole wiskostatin was identified as an antifungal compound,^25^ but is also known to be a potent inhibitor of actin polymerization through inhibition of neural Wiskott-Aldrich syndrome protein (N-WASP) by interaction in a cleft in the regulatory GTPase-binding protein.^27^ Furthermore, wiskostatin has been reported as an inhibitor of DNA synthesis in vaccinia, the prototypical poxvirus.^28^ Carbazole compounds very similar to those reported here have also been identified as inhibitors of the PI3K/Akt/FOXO1a signaling pathway, and have been suggested to target the ATP-binding sites of kinases.^29^ This, together with a general ATPase inhibitory function of the carbazoles in the present work, prompted us to investigate if these carbazoles act as H^+^-ATPase inhibitors by blocking the nucleotide-binding site. Our data suggest that this is not the case, as we observed that a 10-fold increase of ATP did not interfere with the inhibitory activity of the compound. The binding site of the carbazole in the H^+^-ATPase is more likely to be located in the ion inlet cavity of the transmembrane region, where the fungal H^+^-ATPase inhibitor and antimalarial compound KAE609 is also suggested to bind.^30^ The binding cavity surrounded by the transmembrane helices TM1 to TM4 is also a well-known binding pocket for other Ca^2+^-ATPase inhibitors^31^, ^32^ and has also been proposed as the binding pocket for the chemically related tetrahydrocarbazoles, which are also H^+^-ATPase inhibitors with antifungal activity.^33^

Of the compounds evaluated in this study compound **10** was the most potent inhibitor of the H^+^-ATPase in the ATP hydrolysis assay and by far the most potent antifungal compound tested. Within the first ten minutes of exposure to compound **10** fungal cells were potently inhibited in extracellular acidification of the surrounding media, and within thirty minutes a large increase in intracellular ATP was observed. Fungicidal activity was evident after 1 h exposure of **10** (10 µM) in *C. albicans* and 3 h of exposure in *C. glabrata*. Compound **10** and **15** are distinctly more basic than all other compounds tested. Interestingly, due to the molecular features, these compounds also have the highest calculated LogP and lowest LogD values (Supporting Information), which influences membrane permeability and solubility properties. Molecular electrostatic interactions could potentially also benefit from these properties. Compound **10** also differs from **15** by its the possibility to make an intramolecular hydrogen bond in the *N*-1 group, which may greatly facilitate diffusion through membranes. Both compound **10** and carbazole analogues in which R^3^ is substituted H (instead of –OH) appear to be better fungal inhibitors. It would therefore be prudent to evaluate a compound **10** analogue with a substituted H in the R^3^ position in any future antifungal drug development program. Compound **10** was also shown to exhibit potent antibacterial activity and has previously been reported as a bactericidal agent, where it was found to exhibit membrane damaging activity in the bacterial species *Pseudomonas aeruginosa.*^34^, ^35^

In conclusion, our findings suggest that inhibition of the fungal H^+^-ATPase contributes at least partially to the antifungal mechanism of action of these carbazole compounds. Longer periods of fungal cell exposure to compound **10** (>30 min) could potentially compromise the fungal membrane, as has been seen for bacterial membranes.^34^, ^35^ However, further studies are needed to address whether the carbazoles presented here, are in fact able to compromise the fungal membrane. Amphotericin B is a well-known antifungal agent targeting the fungal membrane,^36^ and the development of membrane-targeting agents is an emerging strategy for treating persistent bacterial infections.^37^ Taking this into account, carbazoles may represent a promising scaffold for the development of new anti-infective therapies, as suggested by others.^32^ Likewise, the fungal H^+^-ATPase has long been recognized as a potential promising new antifungal target,^21^ and any future drug discovery program using the carbazoles to target the H^+^-ATPase should pay special attention to the potentially membrane-compromising properties and the unspecific ATPase inhibitory activity of the carbazoles in general.
