# Supplementary material for: Elucidation of antimicrobial activity and mechanism of action by N-substituted carbazole derivatives
Source: Bioorg Med Chem Lett. 2017 Oct 1;27(19):4564–70. doi: 10.1016/j.bmcl.2017.08.067 (PMC5609566; doi:10.1016/j.bmcl.2017.08.067)
Supplement: Supplementary data [file mmc1.docx]

**Supporting Information**

**Materials and methods**

**Chemistry**

Compounds **1 - 4** were part of an acquired screening library. The compounds may be sourced commercially and are identified with their respective CAS numbers: Compound **1** = 91324-15-1; Compound **2** = 263745-17-1; Compound **3** = 69694-78-6; Compound **4** = 304893-76-3. Compound 5 – 19 was synthesis by Aurigene Discovery Technologies Limited, India.

Synthesis of **Compounds 5 - 8, 10 – 12** (General procedure)

A solution of I (1 mM), A (1.5 mM) in 2-propanol (5 mL) was heated at 100 ^o^C under stirring for 12 h in a seal tube. Solvent was removed in vacuo and added with cold water. The precipitated solid was filtered off and was purified by flash column chromatography to afford the desired product.

**Compound 5** (1-(3,6-dichloro-9H-carbazol-9-yl)-3-(1H-imidazol-1-yl)propan-2-ol)

^1^H NMR (400M Hz, DMSO-d_6_) δ 8.32 (s, 2H), 7.67 (d, *J* = 8.8 Hz, 2H), 7.51 (d, *J* = 8.8 Hz, 2H), 7.21 (s, 1H), 6.9 (s, 1H), 5.38 (d, *J* = 6.0 Hz, 1H), 4.41 (d, *J* = 4.0 Hz, 1H), 4.38 (d, *J* = 4.0 Hz, 1H), 4.20 (s, 1H), 4.06 (t, *J* = 8.0 Hz, 2H). MS (ESI) m/z 362.0 (M + 2)^+^.

**Compound 6** (1-(3,6-dichloro-9H-carbazol-9-yl)-3-(1H-1,2,4-triazol-1-yl)propan-2-ol)

^1^H NMR (400M Hz, DMSO-d_6_) δ 8.45 (s, 2H), 7.98 (s, 1H), 7.67 (d, *J* = 8.8 Hz, 2H), 7.51 (d, *J* = 8.8 Hz, 2H), 5.43 (s, 1H), 4.53 (d, *J* = 3.6 Hz, 1H), 4.49 (d, *J* = 4.8 Hz, 1H), 4.41 (d, *J* = 4.8 Hz, 1H), 4.27 (s,1H). MS (ESI) m/z 360.9 (M + 1)^+^.

**Compound 7** (1-(3,6-dichloro-9H-carbazol-9-yl)-3-morpholinopropan-2-ol)

^1^H NMR (400M Hz, DMSO-d_6_) δ 8.31(s, 2H), 7.68 (d, *J* = 8.8 Hz, 2H), 7.50 (d, *J* = 8.8 Hz, 2H), 4.98 (s, 1H), 4.47 (d, *J* = 4.0 Hz, 1H), 4.43 (d, *J* = 3.6 Hz, 2H), 4.31 (d, *J* = 6.4 Hz, 1H), 4.27 (d, *J* = 6.4 Hz, 1H), 4.00 (s, 1H), 3.56 (t, *J* = 4.8Hz, 4H), 2.38-2.31(m, 6H). MS (ESI) m/z 378.9 (M + 1)^+^.

**Compound 8** (1-(3,6-dichloro-9H-carbazol-9-yl)-3-(4-methylpiperazin-1-yl)propan-2-ol)

^1^H NMR (400M Hz, DMSO-d_6_) δ 8.31(s, 1H), 7.67 (d, *J* = 8.8 Hz, 2H), 7.48 (d, *J* = 8.8 Hz, 2H), 4.90 (d, *J* = 4.8 Hz, 1H), 4.47 (d, *J* = 4.0 Hz, 1H), 4.43 (d, *J* = 3.6 Hz, 1H), 4.31 (d, *J* = 6.4 Hz, 1H), 4.27 (d, *J* = 6.4 Hz, 1H), 4.00 (s, 1H), 2.37-2.27 (m, 10H), 2.1 (s, 1H). MS (ESI) m/z 391.9 (M + 1)^+^.

**Compound 10** (1-(sec-butylamino)-3-(3,6-dichloro-9H-carbazol-9-yl)propan-2-ol)

^1^H NMR (400M Hz, DMSO-d_6_) δ 8.31 (s, 2H), 8.69 (d, *J* = 8.8 Hz, 2H), 7.50 (d, *J* = 8.8 Hz, 2H), 5.00 (s,1H), 4.43 (d, *J* = 6.4 Hz, 1H), 4.30 (d, *J* = 6.8 Hz, 1H), 3.90 (s, 1H), 2.50-2.44 (m, 2H), 1.59 (s, 1H), 1.42-1.38 (s, 2H), 1.37 (s, 2H), 0.96 (d, *J* = 3.6 Hz, 3H), 0.86 (d, *J* = 4.8 Hz, 3H). MS (ESI) m/z 365.1 (M + 1)^+^.

**Compound 11** (1-(3,6-dimethoxy-9H-carbazol-9-yl)-3-(4-methylpiperazin-1-yl)propan-2-ol)

^1^H NMR (400 MHz, DMSO-d_6_) δ 7.69 (s, 2H), 7.47 (d, *J* = 8.8 Hz, 2H), 7.03 (d, *J* = 8.8 Hz, 2H), 4.39-4.34 (m, 1H), 4.20-4.15 (m, 1H), 4.01-3.99 (m, 1H), 3.84 (s, 6H), 2.50-2.25 (m, 10H), 2.15 (s, 3H). MS (ESI) m/z 384.0 (M + 1) ^+^.

**Compound 12** (9-(2-hydroxy-3-(4-methylpiperazin-1-yl)propyl)-9H-carbazole-3,6-dicarbonitrile)

^1^H NMR (400M Hz, DMSO-d_6_) δ 8.84 (s, 2H), 7.94 (d, *J* = 8.8 Hz, 2H), 7.88 (d, *J* = 8.8 Hz, 2H), 4.96 (s, 1H), 4.59 (d, *J* = 3.6 Hz, 1H), 4.55 (d, *J* = 3.6 Hz, 1H), 4.43 (t, *J* = 5.6 Hz, 1H), 4.01 (s, 1H), 2.4-2.28 (m, 10H), 2.13 (s, 1H). MS (ESI) m/z 374.9 (M + 1)^+^.

**Compound 9** (1-(3-(3,6-dichloro-9H-carbazol-9-yl)-2-hydroxypropyl)piperidine-4-carboxylic acid)

Methyl piperidine-4-carboxylate (1 mM) in ethanol (5 mL) was added with sodium hydroxide (1 mM) and stirred at room temperature for 5 minutes. I (1 mM) was added and reaction was heated at 80 ^o^C for 12 h under stirring. Reaction mass was poured in to water, extracted with ethyl acetate. The organic layer was dried over anhydrous sodium sulfate and concentrated in vacuo. The residue was purified by flash column chromatography to afford the desired product.

^1^H NMR (400 MHz, DMSO-d_6_) δ 8.31 (s, 1H), 7.61 (d, *J* = 8.8 Hz, 2H), 7.48 (d, *J* = 8.8 Hz, 2H), 4.97 (s, 1H), 4.43 (d, *J* = 3.6 Hz, 1H), 4.31 (d, *J* = 6.8 Hz, 1H), 4.02 (s, 1H), 2.91-2.75 (m, 2H), 2.32-2.27 (m, 2H), 2.10-1.98 (m, 3H), 1.75-1.58 (m, 4H). MS (ESI) m/z 419.4 (M - 1)^-^.

**Compound 13** (9-(2-hydroxy-3-(4-methylpiperazin-1-yl)propyl)-9H-carbazole-3,6-diol)

To a 0 ^o^C cooled solution of III (1 mM) in DCM (10 mL) was added dropwise BBr_3_ (1M in DCM, 2 mM) and the mixture was stirred at same temperature for 2 h. After completion, solvent was removed in vacuo; diluted with ethyl acetate and washed with sat. bicarbonate followed by water and brine. The organic layer was dried over anhydrous sodium sulfate and concentrated in vacuo. The residue was purified by flash column chromatography to afford the desired product.

^1^H NMR (400 MHz, DMSO-d_6_) δ 8.83 (s, 2H), 7.30 (m, 4H), 6.86 (dd, *J* = 8.8, 2.4 Hz, 2H), 4.82 (br s, 1H), 4.31-426 (m, 1H), 4.12-4.08 (m, 1H), 3.97 (br s, 1H), 2.50-2.24 (m, 10H), 2.15 (s, 3H). MS (ESI) m/z 356.0 (M + 1)^+^.

Synthesis of Compounds **14 – 19** (General procedure)

Amine A (1.2 mM) in DMF (5 mL) was added with potassium carbonate (1.2 mM) and stirred at room temperature for 5 minutes. II (1 mM) was added and reaction mixture was heated at 50 ^o^C for 8 h. Reaction mass was poured in to water, extracted with ethyl acetate. The organic layer was dried over anhydrous sodium sulfate and concentrated in vacuo. The residue was purified by flash column chromatography to afford the desired product.

**Compound 14** (3-(3,6-dichloro-9H-carbazol-9-yl)-N,N-dimethyl-propan-1-amine)

^1^H NMR (400 MHz, DMSO-d_6_) δ 8.33 (s, 2H), 7.68 (d, *J* = 8.8 Hz, 2H), 7.50 (d, *J* = 8.8 Hz, 2H), 4.43 (t, *J* = 6.4 Hz, 2H), 2.16 (t, *J* = 7.2 Hz, 2H), 2.50 (s, 6H), 1.75 (t, *J* = 7.6 Hz, 2H). MS (ESI) m/z 321.0 (M + 1)^+^.

**Compound 15** ((3-(3,6-dichloro-9H-carbazol-9-yl)-N,N-dimethyl-butyl-1-amine)

^1^H NMR (400 MHz, DMSO-d_6_) δ 8.33 (s, 2H), 7.68 (d, *J* = 8.8 Hz, 2H), 7.50 (d, *J* = 8.8 Hz, 2H), 4.42 (t, *J* = 6.8 Hz, 2H), 2.16 (t, *J* = 7.2 Hz, 2H), 2.50 (s, 6H), 1.75 (t, *J* = 7.6 Hz, 2H), 1.39 (t, *J* = 7.2 Hz, 2H). MS (ESI) m/z 336.1 (M + 1)^+^.

**Compound 16** (3,6-dichloro-9-(3-(4-methylpiperazin-1-yl)propyl)-9H-carbazole)

^1^H NMR (400 MHz, DMSO-d_6_) δ 8.33 (s, 2H), 7.68 (d, *J* = 8.8 Hz, 2H), 7.50 (d, *J* = 8.8 Hz, 2H), 4.43 (t, *J* = 6.4 Hz, 2H), 2.30-2.17 (m, 12H), 1.90 (t, *J* = 6.4 Hz, 2H). MS (ESI) m/z 376.2 (M + 1)^+^.

**Compound 17** (3,6-dichloro-9-(3-(4-methylpiperazin-1-yl)propyl)-9H-carbazole)

^1^H NMR (400 MHz, DMSO-d_6_) δ 8.33 (s, 2H), 7.68 (d, *J* = 8.8 Hz, 2H), 7.50 (d, *J* = 8.8 Hz, 2H), 4.43 (t, *J* = 6.4 Hz, 2H), 2.37-2.10 (m, 12H), 1.75 (t, *J* = 6.8 Hz, 2H), 1.42 (t, *J* = 7.2 Hz, 2H). MS (ESI) m/z 390.0 (M + 1)^+^.

**Compound 18** (4-(3-(3,6-dichloro-9H-carbazol-9-yl) propyl)morpholine)

^1^H NMR (400 MHz, DMSO-d_6_) δ 8.33 (s, 2H), 7.68 (d, *J =* 8.8 Hz, 2H), 7.50 (d, *J* = 8.8 Hz, 2H), 4.43 (t, *J* = 6.8 Hz, 2H), 3.51 (t, *J* = 4.4 Hz, 4H), 2.24 (t, *J* = 7.2 Hz, 3H), 1.77 (t, *J* = 7.6 Hz, 2H). MS (ESI) m/z 363.1 (M + 1)^+^.

**Compound 19** (4-(3-(3,6-dichloro-9H-carbazol-9-yl) butyl)morpholine)

^1^H NMR (400 MHz, DMSO-d_6_) δ 8.33 (s, 2H), 7.68 (d, *J* = 8.8 Hz, 2H), 7.50 (d, *J* = 8.8 Hz, 2H), 4.43 (t, *J* = 6.4 Hz, 2H), 3.51 (t, *J* = 4.4 Hz, 4H), 2.24 (t, *J* = 7.2 Hz, 3H), 1.77 (t, *J* = 7.6 Hz, 2H), 1.43 (t, *J* = 7.6 Hz, 2H). MS (ESI) m/z 377.0 (M + 1)^+^.

**Table 1: LogP, LogD and pKa values (ACDLabs Build 2726):**

| Compound | LogP | LogD7.4 | pKa |
| --- | --- | --- | --- |
| 7 | 4.7 | 4.6 | 6.6 |
| 8 | 4.6 | 4.0 | 7.6 |
| 10 | 5.9 | 3.6 | 9.2 |
| 15 | 5.7 | 3.4 | 9.7 |
| 16 | 5.0 | 4.3 | 7.7 |
| 17 | 5.2 | 4.5 | 7.8 |

**Protein preparation**

Pma1-containing plasma membranes were prepared from *S. cerevisiae* RS72 yeast cells that had been transformed with the full-length cDNA of the *S. cerevisiae* plasma membrane H^+^-ATPase isoform PMA1 under control of the PMA1 promoter, using the same procedure as described in Kongstad *et al.*, 2014^1^. For the preparation of *C. albicans* membranes, a 100 ml overnight culture grown in YPD (10 g/L yeast extract (BD, Sparks, MD), 20 g/L Bacto peptone (BD), 20 g/L D-(+)-glucose) was transferred to 1 L YPD and grown for a further 7 h at RT with agitation (150 rpm), followed by cell harvesting and subsequent membrane preparation using the same procedure as described in Kongstad *et al.*, 2014^1^. Further plasma membrane purification by sucrose gradient sedimentation did not improve the purity of the *C. albicans* microsomal preparations and this step was therefore omitted. Rabbit sarcoplasmic reticulum membranes containing Sarco/Endoplasmic Reticulum Ca^2+^ ATPase (SERCA) were kindly provided by Dr. Claus E. Olesen and Dr. Jesper V. Møller, Aarhus University and prepared as described in Andersen *et al.*, 1985^2^. Pig kidney Na^+^,K^+^-ATPase was kindly provided by Dr. Natalya Fedosova, Aarhus University and prepared as described in Klodos *et al.*, 2002^3^.

**Colorimetric ATPase assay**

The compound libraries were purchased from Enamine Ltd, Ukraine and Chembridge Corp, USA. ATPase activity was determined by measuring the amount of liberated phosphate from ATP hydrolysis. Reactions, including protein membrane preparation (0.1 to 2.5 µg per assay) and various concentrations of exogenously added compounds, were conducted in the following buffers: Pma1 buffer: 17.5 mM MOPS-NaOH pH 7, 7 mM MgSO_4_, 44 mM KNO_3_ (vacuolar ATPase inhibitor), 22 mM NaN_3_ (mitochondrial ATPase inhibitor), 0.22 mM Na_2_MoO_4_ (acid phosphatase inhibitor); SERCA buffer: 9 mM MOPS-NaOH pH 7, 2.7 mM MgCl_2_, 0.1 µM CaCl_2_ and 72 mM KCl; Na^+^,K^+^-ATPase buffer: 30 mM MOPS-NaOH pH 7, 40 mM NaCl, 4 mM MgCl_2_ and 20 mM KCl. Reactions were initiated by the addition of Na-ATP to a final concentration of 2.5 mM (Pma1 and Na^+^,K^+^-ATPase) or 5 mM (SERCA), followed by 30 minutes incubation at 30 ˚C. The amount of liberated phosphate was determined colorimetrically after addition of STOP solution (mixture of L-ascorbic acid, ammonium heptamolybdate tetrahydrate, and HCl to give final concentrations of 65 mM, 2.2 mM, and 189 mM, respectively) with 5 minutes incubation at room temperature (RT) followed by addition of arsenite solution (mixture of NaAsO_2_, sodium citrate dihydrate, and acetic acid to give final concentrations of 3.1 mM, 28 mM, and 141 mM, respectively). Absorption was measured at 860 nm after an additional 30 minutes incubation at RT.

**NADH-coupled ATPase assay**

The initial rate of ATP turnover at RT was measured spectrophotometrically at 340 nm by an NADH-coupled ATPase assay that uses phosphoenolpyruvate to regenerate ATP, as described in Møller *et al.*, 1980^4^. The reaction buffer contained 17.5 mM MOPS-NaOH pH 7, 44 mM KNO_3_, 22 mM NaN_3_, 0.22 mM Na_2_MoO_4_, 1 mM phosphoenolpyruvate, 0.27 mM NADH, 83 µg/ml pyruvate kinase, 22 µg/ml L-lactate dehydrogenase, either 1, 5 or 10 mM ATP, and MgSO_4_ to give a Mg^2+^ concentration of 2 mM in excess over the ATP concentration. The ATPase reaction was initiated by the addition of 0.2-0.3 mg/ml of *S. cerevisiae* Pma1-containing membranes followed by addition of compound (resulting in a final DMSO concentration of 1.1%).

**Fungal growth inhibition**

Compound hits from the library screening were evaluated against baker yeast *S. cerevisiae* and *C. albicans* (ATCC 90028). The minimum inhibitory concentration (MIC) was defined as the lowest concentration inhibiting visual growth of the microorganism and this was determined in triplicate. Standard errors were typically less than 5%. MICs for synthesized compounds were determined on the following fungal isolates: *Saccharomyces cerevisiae* (ATCC 9763), *Candida albicans* (SC5314), *Candida krusei* (ATCC 6258), *Candida glabrata* (ATCC 90030), *Aspergillus flavus* (ATCC MYA-1005) and *Aspergillus fumigatus* (ATCC 13073), which were purchased from American Type Culture Collection, and *C. glabrata* strain Cg003, which was kindly provided by Julius Subik, Comenius University in Bratislava, Slovak Republic. Cg003 is a clinical isolate, characterized to be resistant to fluconazole and itraconazole due to overexpression of the multidrug resistance efflux pumps Cdr1p and Cdr2p^5^. The fungal growth assay was performed as described in Kjellerup *et al*., 2017^6^. The MFC was the minimum concentration that resulted in no colony forming units (CFU) and was determined after MIC determination by plating 5 µL of the mixture from wells with no visible growth onto YPD agar plates followed by 24 h incubation at 30 °C.

**Time-kill assay**

*C. albicans* (SC5314) and *C. glabrata* (ATCC 90030) cells (1 x 10^5^ CFU/ml) were incubated in 10 mL RPMI media at 30 °C with gentle agitation (150 rpm) in the presence of amphotericin B (1 µM) or compound **10** at a concentration of 5 or 10 µM. At the indicated time points (0, 1, 3, 5 and 24 h at 30 °C incubation, 150 rpm), 100-μL aliquots were removed, serially diluted (10-fold) in saline (0.9% NaCl), and plated on YPD agar plates. The resulting CFUs were enumerated after incubation at 30 °C for 48 h. Cells treated with DMSO (1%, v/v) served as a control.

**Bacterial growth inhibition assay**

The bacterial strains used in this study were purchased from DSMZ, Germany. Bacteria cells were grown in Mueller-Hinton broth (MHB) to mid-exponential phase and freeze stocks were prepared at 1-2 × 10^8^ CFU/mL in MHB and 20% glycerol and stored at -20˚C. These stocks were used to inoculate bacterial growth assays at a final concentration of 5 × 10^5^ CFU/mL in MHB together with 1.5 % DMSO or compound in a 96-well plate and incubated for 16-29 h at 35˚C, whereafter growth was determined by reading at OD_600_. Kanamycin and ampicillin were used as positive control compounds. Experiments were performed at least twice and standard errors were typically less than 5 %.

**Intracellular ATP determination**

*C. albicans* cells were transferred to 3 ml of YPD media and grown overnight at 30 °C and 150 rpm. The cells were pelleted at 2000 × *g* for 2 min. and washed in PBS (Sigma-Aldrich, catalog number 8537) twice before re-suspension to an OD_600_ of 0.1. Cell suspension (98 µL) and 2 µl compound in DMSO were incubated for 30 minutes at RT. Forty microliters of this suspension was then transferred to a black 96-well plate containing 40 µl BacTiterGlo reagent (Promega, Madison, WI) and the plate was incubated for 15 minutes in the dark at room temperature. Luminescence was read on a SpectraMax X5 (Molecular Devices, Sunnyvale, CA) plate reader with 10 s shake and 0.5 s integration time. A standard curve containing 10, 100 and 1000 nM ATP was prepared for every experiment.

**Extracellular acidification assay**

The ability of *S. cerevisiae* and *C. albicans* to acidify the media upon glucose addition was evaluated using the pH sensor dextran (40,000MW)-fluorescein-isothiocyanate as described in Kjellerup *et al.,* 2017^6^. Briefly, the cells was starved overnight in 50 mM KCl, pH 6.7 at 4 °C. The cell suspension (Final OD_600_ of 0.19) was mixed with 1.3 µg/ml dextran (40,000MW)-fluorescein-isothiocyanate, 1.5 % DMSO containing inhibitors and the assay initiated with the addition of 2 % D-(+)-glucose. The rate of media acidification was calculated based on the slope of the fluorescent drop in the first 12 minutes after glucose addition.

**References**

1. Kongstad, K. T.; Wubshet, S. G.; Johannesen, A.; Kjellerup, L.; Winther, A. L.; Katharina, A.; Staerk, D. *J. Agric. food Chem.* **2014**, *62*, 5595–5602.

2. Andersen, J. P.; Lassen, K.; Møller, J. V *J. Biol. Chem.* **1985**, *260*, 371–80.

3. Klodos, I.; Esmann, M.; Post, R. L. *Kidney Int.* **2002**, *62*, 2097–2100.

4. Møller, J. V; Lind, K. E.; Andersen, J. P. *J. Biol. Chem.* **1980**, *255*, 1912–1920.

5. Berila, N.; Subik, J. *Mycopathologia* **2010**, *170*, 99–105.

6. Kjellerup, L.; Gordon, S.; Cohrt, K. O.; Brown, W. D.; Fuglsang, A. T.; Winther, A.-M. L. *Antimicrob. Agents Chemother.* **2017**, AAC.00032-17.
